# Exercise in Hypertrophic Cardiomyopathy: Recent Conceptual Changes and Recommendations for Pre-Exercise Tests

**DOI:** 10.31083/j.rcm2406166

**Published:** 2023-06-06

**Authors:** Jiwon Seo, Eui-Young Choi, Se-Joong Rim

**Affiliations:** ^1^Division of Cardiology, Department of Internal Medicine, Gangnam Severance Hospital, Yonsei University College of Medicine, 06273 Seoul, Republic of Korea

**Keywords:** hypertrophic cardiomyopathy, exercise, sudden cardiac arrest, exercise stress echocardiography

## Abstract

Traditionally, individuals with hypertrophic cardiomyopathy (HCM) have been advised not to participate in more than low-intensity exercises. HCM was originally described in the context of sudden death, and early literature from the registry showed that HCM was the most common cause of sudden cardiac death in young athletes. Therefore, there has long been a concern that exercise could trigger ventricular arrhythmia and sudden cardiac death. Although a few patients with HCM may progress along deteriorating disease pathways, many have no clinically significant symptoms or adverse events, no need for major treatment, and a normal life expectancy. Therefore, the routine restriction of any exercise intensity in this large group deprives them of the multiple benefits of exercise and may have detrimental effects on long-term clinical outcomes. However, it has been reported that light to moderate exercise is acceptable for many patients with HCM, and recent evidence suggests that vigorous exercise does not increase the risk of sudden death in this population. Thus, we reviewed previous literature regarding the effects of exercise in patients with HCM and provided cutting-edge information on the safety and concerns of exercise. In addition, based on our experience and previous research, we reviewed the conditions that should be met before starting exercise and the tests required to confirm them.

## 1. Introduction 

For many people, exercise has myriad benefits for their mental and physical health. However, in some conditions or types of heart disease, including hypertrophic cardiomyopathy (HCM), there has been a concern that exercise could trigger sudden cardiac death. Traditionally, these populations have been discouraged from participating in competitive sports. HCM is one of the most common genetic heart diseases, affecting approximately 1 in 500 people worldwide [1, 2]. HCM is characterized by left ventricular (LV) hypertrophy of various morphologies with many clinical manifestations such as arrhythmia, mitral regurgitation, syncope, heart failure, myocardial ischemia, and sudden cardiac death. However, in most patients with HCM, LV hypertrophy is not progressive, and only 1% of the annual mortality has been reported in non-referral cohorts. Although a few patients with HCM may progress along deteriorating disease pathways, many have no clinically significant symptoms or adverse events, no need for major treatment, and a normal life expectancy [3]. Rarely-occurred sports-related sudden cardiac deaths can be avoided by restricting physical exercises in these patients [4]; however, routine restriction of exercise intensity in this large group deprives them of the benefits of proper exercise and may have detrimental effects on long-term clinical outcomes. Certainly, the safe level of activity for patients with HCM remains controversial; however, recent data on exercise in patients with HCM have accumulated, and a new consensus has emerged, which differs from traditional exercise restrictions [5, 6].

Thus, we reviewed previous evidence regarding the effects of exercise and provided cutting-edge information on the safety and concerns of exercise in patients with HCM. In addition, little is known about tests that might be useful before starting an exercise in patients with HCM. We have reported the hemodynamics and myocardial function during exercise in patients with HCM on exercise stress echocardiography [7, 8]; here, we suggest the use of particular tests as helpful tools in pre-exercise evaluation.

## 2. Concerns about Exercise in HCM Patients

### 2.1 Can Exercise Cause or Worsen Cardiac Symptoms? 

**Fig. 1.**
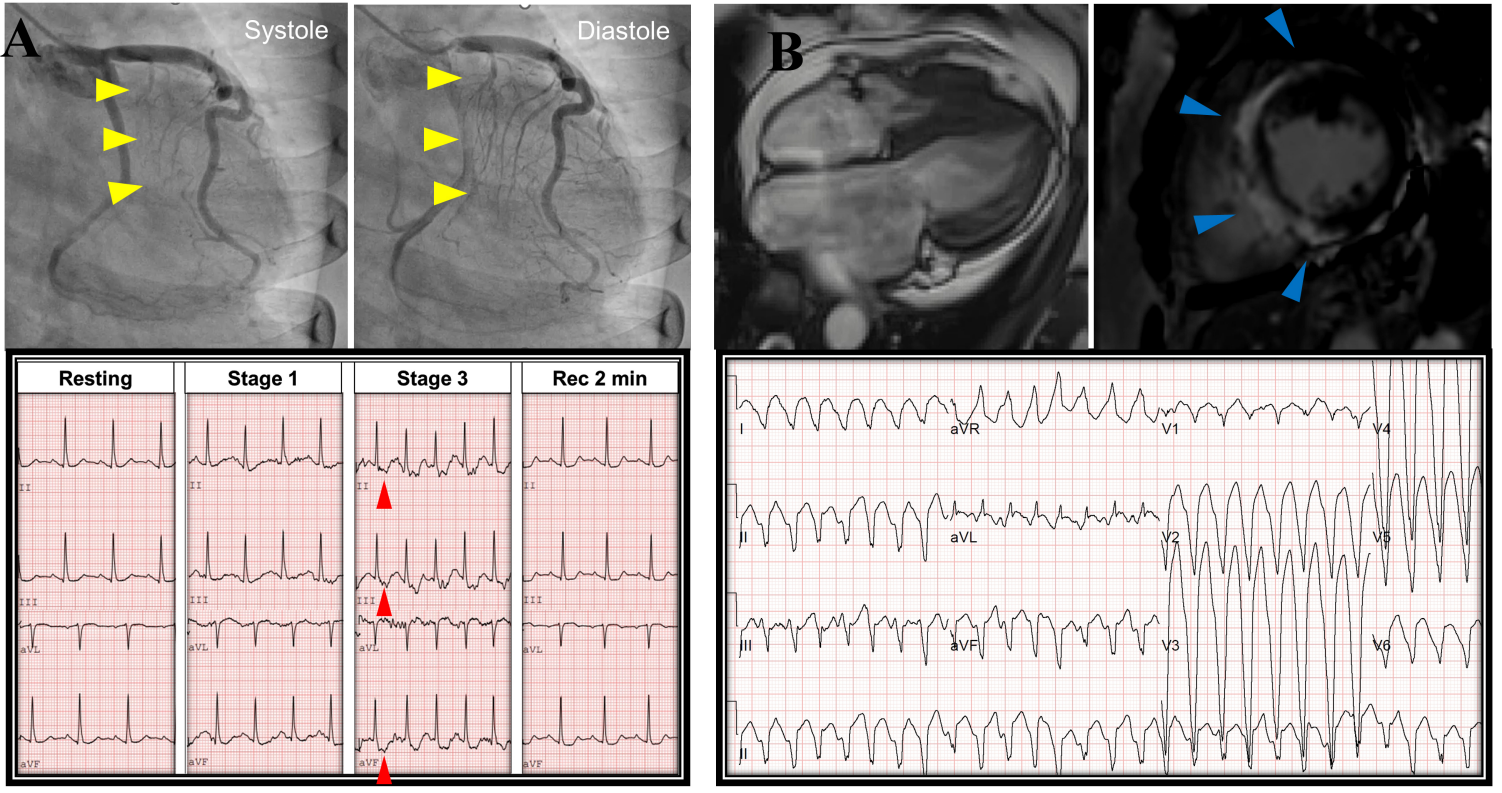
**Representative case for hypertrophic cardiomyopathy**. (A) Case of compressive systolic deformation of the coronary arteries (yellow arrowheads) and ST depression during in Stage 3 of Bruce protocol treadmill test (red arrowheads). (B) Another case of extensive late gadolinium enhancement (blue arrowheads) and exercise-induced ventricular tachycardia.

While many patients with HCM are asymptomatic, others develop HCM-related symptoms such as chest pain, dyspnea on exertion, syncope, and palpitations [9]. These symptoms can be triggered or exacerbated by exercise. Angina is a relatively common symptom that occurs in 25–50% of patients with HCM and may be persistent at rest; similarly, it can be provoked by exertion. Angina without coronary occlusion in HCM might be due to microvascular ischemia, increased oxygen demand in the hypertrophied myocardium, coronary flow abnormalities, or myocardial bridging, usually exacerbated by exercise [10, 11, 12]. Fig. 1A presents a typical case of compressive systolic deformation of the coronary arteries and ST depression during a treadmill test that caused angina during exercise. Syncope is a symptom that occurs during or immediately after exertion. For example, approximately 15–60% of patients with HCM reported at least one syncope or presyncope episode [13]. Some complex mechanisms trigger the development of this condition, such as aggravating LV outflow tract (OT) obstruction, ventricular baroreflex activation with inappropriate vasodilatation, and myocardial ischemia [14]. During or immediately after peak exercise, systemic vascular resistance failed to increase or paradoxically decreased in HCM, whereas it increased in normal controls [15]. Moreover, the highest peak left ventricular outflow tract (LVOT) pressure gradient may occur immediately after exercise, even in nonobstructive HCM [8]. These conditions lead to abnormal blood pressure responses during exercise and may contribute to syncope in HCM [15].

### 2.2 Can Exercise Worsen LV Hypertrophy or Trigger Ventricular Arrhythmia?

A notable concern regarding intensive exercise in patients with HCM is worsening myocardial hypertrophy. Although athletic heart, bradycardia, cardiomegaly, and cardiac hypertrophy without any symptoms are common non-pathological conditions in athletes, theoretically, they could further promote LV hypertrophy in individuals with HCM. In addition, particularly when these concerns arise in genotype-positive and phenotype-negative individuals, these patients have been described as having preclinical HCM. However, there is no clear evidence that chronic exercise worsens LV hypertrophy in phenotype-positive patients or triggers hypertrophy in them. Moreover, sudden death in these individuals is rare; therefore, the current consensus is to not specifically restrict exercise to genotype-positive and phenotype-negative patients.

Finally, HCM is believed to be the most common cause of sudden cardiac death in the athletic population and was originally described in the context of sudden death. The overall sudden cardiac death rate in adult patients with HCM is low (0.43%), and contemporary HCM-related sudden cardiac death rates have been reduced (0.32%/year), representing a 2-fold decrease compared with prior-treatment eras [1]. However, sudden death remains the most prominent manifestation of HCM owing to early literature from the registry showing that HCM was the most common cause of sudden cardiac death in young athletes; the events were often broadcast vividly in the media. Conversely, ventricular tachyarrhythmia is the leading cause of sudden cardiac death in patients with HCM. Although the mechanisms of arrhythmogenicity in HCM are merely understood, myocyte disarray and fibrosis in the hypertrophied myocardium may be the substrates for arrhythmias (Fig. 1B) [16]. Similarly, LVOT obstruction, increased vagal tone, myocardial ischemia, and vigorous exercise can trigger ventricular tachycardia/ventricular fibrillation with these substrates [17]. This has led to HCM being considered a precautionary factor for disqualifying young athletes. In contrast, more recent statistics on the risk of sudden cardiac death differ from expectations. HCM accounts for ˂10% of all sudden cardiac deaths [18, 19], and it is attributed to a far lower proportion of sudden cardiac deaths in the statistics of athlete associations in the United States [20]. In a multinational sports safety registry for athletes with an implantable cardiac defibrillator (ICD), although more patients experienced appropriate and inappropriate ICD shocks during exercise, there was no difference in the rates of ICD shocks between usual physical activities and competitive sports [21, 22]. These findings suggest that there are myths about the high risk of exercise-induced sudden cardiac death in patients with HCM and that mild to moderate exercise can be considered in most HCM populations. Furthermore, more liberal recommendations can be suggested for competitive exercises for individuals considered at low risk.

## 3. Current Guidelines and Consensus on Exercise in Patients with HCM

### 3.1 For Competitive Athletes with HCM

The 2015 statement by the American Heart Association/American College of Cardiology maintained an early conservative stance. They suggested that athletes with the disease phenotype of LV hypertrophy could only participate in low-intensity sports and should not participate in the most competitive sports. Table 1 shows that the classification of sports as defined in the 2015 guidelines according to exercise intensity expressed as percentage of maximal oxygen uptake [23].

**Table 1.** **Classification of sports according to exercise intensity**.

	Example exercise
Low intensity (${\mathrm{VO}}_{2}$ max <50%)	Bowling, cricket,curling, golf, riflery, yoga
	Archery, auto racing, diving, equestrian, motorcycling
	Bobsledding, luge, field events (throwing), gymnastics, martial arts, rock climbing, sailing, water skiing, weightlifting, windsurfing
Moderate intensity (${\mathrm{VO}}_{2}$ max 50–75%)	Baseball, softball, fencing, table tennis, volleyball
	American football, field events (jumping), rodeoing, rugby (adapted), sprint running, surfing, synchronized swimming, ultra racing
	Bodybuilding, downhill skiing, skateboarding, snowboarding, wrestling
High intensity (${\mathrm{VO}}_{2}$ max >75%)	Badminton, classic technique cross-country skiing, field hockey, orienteering, race walking, racquetball, squash, long-distance running, soccer
	Basketball, ice hockey, skating technique cross-country skiing, lacrosse, middle-distance running, swimming, team handball, tennis
	Boxing, canoeing, kayaking, cycling, decathlon, rowing, speed skating, triathlon

Exercise intensity expressed as percentage of maximal oxygen uptake, low (<50%), moderate (50–75%), high (>75%).${\mathrm{VO}}_{2}$ max, maximal oxygen consumption.

This recommendation was independent of all clinical circumstances, including age, genotype, LVOT obstruction, and whether septal reduction therapy was performed [24]. However, this statement has changed significantly in the 2020 Guidelines for diagnosing and treating HCM. According to this recommendation, participation in moderate- and high-intensity competitive sports may be deemed appropriate after a comprehensive assessment and shared discussion with experts [25]. A similar suggestion was made in the European Society of Cardiology (ESC) Guidelines for Sports Cardiology and Exercise, published in the same year. They recommended that those with no markers of increased risk (e.g., cardiac symptoms or history of cardiac arrest, unexplained syncope, moderate or high ESC risk score, abnormal blood pressure response to exercise, high LVOT gradient at rest, or exercise-induced arrhythmia) should be considered for participation in all competitive sports following expert assessment [26]. Conversely, the prohibition of ICD implantation to participate in sports remained unchanged.

These guidelines introduced the concept of shared decision-making regarding sports participation in HCM. However, quantifying accurate risk prediction for sports participation is challenging and may vary across many physical activities required by different sports. Therefore, the guidelines describe an opportunity for flexibility, individual responsibility, and choice in the decision-making process regarding eligibility for an athlete with HCM. Currently, there are insufficient data to frame these discussions and inform patient decisions; further high-quality research is needed.

### 3.2 For a General Population with HCM

Early guidelines of American and European cardiology societies recommended that patients with HCM refrain from all but low-intensity sports independent of ICD use [27, 28]. Conversely, recent American and ESC guidelines recommend that mild-to-moderate-intensity recreational exercise improves cardiorespiratory fitness, physical functioning, life quality, and overall health. This is consistent with physical activity guidelines for the general population [25]. They suggested that even individuals with markers of increased risk of participating in moderate-intensity recreational exercise may be considered [26]. A recent prospective large-scale study of 1534 patients with HCM (Lifestyle and Exercise in Hypertrophic Cardiomyopathy [LIVE-HCM] trial) was recently completed. Vigorous exercise, defined as >6 metabolic equivalents of task (METs) for >60 h per year, was not associated with an increased (hazard ratio [HR], 1.01, 90% CI, 0.68–1.48; *p* = 0.98) risk of cardiac events (total mortality, cardiac arrest, ventricular arrhythmia treated with an ICD, or fainting likely due to arrhythmia) [29]. This study suggests that there is no need to routinely restrict vigorous exercise in patients with HCM as part of a shared decision-making framework with consultation with experienced HCM physicians.

## 4. Benefits of Exercise in the HCM Population

The effect of exercise on hypertrophy progression in patients with HCM remains unclear. Theoretically, isotonic exercise can induce physiological hypertrophy, which may be detrimental to HCM. However, physiological and pathological hypertrophy pathways differ *in vitro* and *in vivo * [30]. Moreover, endurance exercise promotes cavity dilation, which could benefit patients with HCM who have LVOT obstruction due to a small LV chamber. For example, a study characterizing the clinical profile of young athletes with HCM showed that athletes with HCM had less LV hypertrophy, larger LV cavities, and normal indices of diastolic function compared with sedentary patients [31]. A similar proportion of athletes with HCM and sedentary patients demonstrated late gadolinium enhancement (LGE), a marker of myocardial fibrosis, on cardiac magnetic resonance (CMR) imaging and exhibited apical hypertrophy [31].

Few studies have examined the benefits of exercise in patients with HCM. For example, a murine model of a myosin mutation found that exercise exerted a protective effect by preventing fibrosis and reducing myocyte disarray [32]. Similarly, in a human study, a preliminary randomized trial showed that moderate-intensity exercise, compared with usual activities, resulted in a statistically significant increase in exercise capacity at 16 weeks in patients with HCM [6]. Furthermore, an observational study showed that increased lifetime vigorous exercise was associated with larger LV volumes and favorable diastolic function in HCM [5]. Additionally, a recent population-based cohort study (n = 7666) suggested that moderate-to vigorous-intensity physical activity is associated with a progressive reduction in all-cause and cardiovascular mortality in a middle-aged population of patients with HCM [33].

In contrast, a survey-based study demonstrated that many participants with HCM had been advised to abstain from exercise; their physical activity was markedly low, and they had a higher body mass index [34]. More than half of patients with HCM failed to meet the minimal physical activity recommendation, and 70% were obese or pre-obese [35]. These lifestyle and metabolic conditions may increase the risk of atherosclerotic cardiovascular diseases, heart failure, and atrial fibrillation [36]. The severity of these conditions is underlined by the significantly lower survival rate, more than a two-fold increased risk of sudden cardiac death in patients with HCM, and concomitant severe coronary artery disease [37].

If the incidence of adverse events from exercise in patients with HCM is much lower than traditionally thought, it would be reasonable to expect a significant improvement in the lifetime health and life quality of patients with HCM as a result of physical activity, similar to the one observed for the general population. However, more evidence is needed to explain how exercise affects patient outcomes and impacts this population’s long-term prognosis.

## 5. Useful Tests before Starting an Exercise

### 5.1 Exercise Stress Echocardiography

Exercise testing should be a part of the routine assessments of functional capacity in patients with HCM who intend to exercise [26]. Exercise stress echocardiography provides more information about the dynamic myocardial structure and function than simple exercise testing. All protocols, including treadmill, supine, or semi-supine bicycle ergometers, can be useful for this purpose in individuals with HCM. Generally, evaluating multiple parameters during peak semi-supine bicycle exercise is technically easier. However, immediately after peak exercise, standing leads to a greater decrease in preload; therefore, if exercise does not produce LVOT obstruction gradients, assessing post-exercise standing position in post-exercise stage should be considered [38]. In one study, postprandial upright stress echocardiography was performed in patients with non-obstructive HCM. In 65% of patients, LVOT gradients >50 mmHg were detected [39]. As most exercise is performed in a standing posture, testing in this posture may be more appropriate to stratify risk prior to exercise. Exercise stress echocardiography provides information on patients with HCM, as shown in Table 2.

**Table 2.** **Information from exercise stress echocardiography in individuals with HCM**.

Exercise-related cardiac symptom
Presence of dynamic LVOT obstruction
Abnormal blood pressure response to exercise
Diastolic and contractile reserve
Significant ST-depression, inducible wall motion abnormalities
Dynamic increase in mitral regurgitation
Monitoring the response to therapy
Exercise-induced arrhythmia

HCM, hypertrophic cardiomyopathy; LVOT, left ventricular outflow tract.

This test evaluated exercise hemodynamics and functional adaptation; provocation tests were performed before exercise [38]. Moreover, the presence of exercise-induced arrhythmia and abnormal blood pressure response to exercise, which are significant features of sudden cardiac death, can also be determined. A recent study showed that abnormal exercise test results (abnormal blood pressure response, significant ST change, or complex ventricular ectopy) were independently associated with lower transplant-free survival in children with HCM [40]. Similarly, exercise-induced ischemia was independently associated with sudden cardiac death [40].

Additionally, most patients with HCM have decreased exercise capacity owing to failure in stroke volume augmentation due to diastolic dysfunction. Therefore, stroke volume augmentation during exercise should be a key target for exercise training in these patients [41]. Furthermore, changes in the diastolic function reserve assessed by exercise stress echocardiography could provide incremental information on exercise capacity [7]. LVOT obstruction is usually combined with the systolic anterior motion of the mitral valve and septal contact due to flow resistance, which also leads to mitral regurgitation. Mitral regurgitation can be exacerbated during exercise in patients with HCM due to increased LV filling pressure, pulmonary artery pressure, and forward stroke volume. This may be another reason for exercise intolerance [42]. Thus, initial assessment of exercise capacity, the setting of exercise goals, and follow-up after exercise-training with exercise stress echocardiography are crucial in HCM. Table 3 presents a summary of previous literature on exercise stress echocardiography in HCM [7, 8, 43, 44, 45, 46, 47, 48, 49, 50, 51].

**Table 3.** **Summary of the literature on the use of exercise stress echocardiography in HCM**.

First author (Year)	Type of exercise test	Individual characteristics	Summary
Okeie (2000) [43]	Supine bicycle	39 patients with HCM but without obstructive symptoms at rest or coronary artery disease	Exercise-induced systolic dysfunction occurred in 50% of patients with HCM. Regional wall motion abnormalities were present in hypertrophied segments
Ha (2006) [44]	Supine bicycle	40 patients with HCM and 41 control subjects	Augmentation of LV longitudinal function during exercise is blunted in patients with HCM
Choi (2008) [7]	Supine bicycle	32 patients with HCM	Diastolic function reserve can provide incremental information for the prediction of exercise capacity
Peteiro (2012) [45]	Treadmill exercise	239 patients with HCM	Exercise capacity and change in wall motion score index during exercise associated with cardiac event
Peteiro (2015) [46]	Treadmill exercise	148 patients with HCM who underwent CMR	Patients with exercise-induced wall motion abnormality are more likely to have abnormal results on CMR (LGE, perfusion defects)
Ciampi (2016) [47]	Treadmill or semi-supine bicycle	608 patients with HCM	Exercise-induced ischemic criteria were associated to worse prognosis
Pozios (2018) [48]	Treadmill exercise	95 patients with HCM and 26 controls	Postexercise strain rate correlates with LGE and exercise capacity. Exercise strain rate predicts ventricular arrhythmia
Wu (2019) [49]	Semi-supine bicycle	76 patients with HCM (48 without and 28 with RV hypertrophy) and 30 age‐ and sex‐matched controls	Patients with HCM have impaired right ventricular mechanics and significantly reduced right ventricular contractile reserve during exercise
E1 Assaad (2020) [50]	Treadmill exercise or upright bicycle	91 children (67% males, median age 12 years) with HCM	Exercise stress echocardiography can be performed safely and served as an effective tool in children (≥6 age)
Kim (2021) [8]	Semi-supine bicycle	35 patients with non-obstructive HCM	Highest peak LVOT pressure gradient predominantly occurred immediately after exercise rather than during peak exercise
Pálinkás (2022) [51]	Treadmill or supine bicycle	128 patients with HCM	B-lines were found in 10% at rest and 30% during exercise. Diastolic impairment and mitral regurgitation were key determinants of pulmonary congestion during exercise

HCM, hypertrophic cardiomyopathy; LV, left ventricular; CMR, cardiac magnetic resonance imaging; LGE, late gadolinium enhancement; RV, right ventricular; LVOT, left ventricular outflow tract.

### 5.2 Holter Monitoring

Current guidelines recommend 24- to 48-hour ambulatory electrocardiographic monitoring to identify patients at risk for sudden cardiac death. This guides managing arrhythmias because episodes of non-sustained ventricular tachycardia (NSVT) are significantly associated with a higher risk of sudden cardiac death, particularly in younger individuals with HCM. In addition, some studies have shown that the presence of NSVT on Holter monitoring is significantly associated with elevated serum troponin levels, indicating myocardial damage [52, 53]. Recently, continuous electrocardiogram (ECG) monitoring has been actively used. The process involves using wearable devices that provide more prolonged monitoring of heart rhythms. Although prolonged heart rhythm monitoring helps diagnose more episodes of NSVT, there were difficulties in evaluating the risk for sudden cardiac death due to too frequent detection of NSVT (75%) [54]. It was reported that a longer, complex, and multifocal origin is associated with a higher incidence of appropriate shock in patients with ICD [55]. Hence, the efficacy of prolonged heart rhythm monitoring using contemporary devices needs further evaluation.

### 5.3 Other Potentially Useful Tests

CMR is a non-invasive imaging modality that provides detailed information on the structure and function of the heart. In addition, CMR can provide information on the presence of an LV apical aneurysm and the extent of LGE, which are closely associated with sudden cardiac death [56, 57, 58]. However, an optimal quantification method remains unestablished, and whether exercise intensity should be modified according to different extents of LGE measured using CMR has not been studied.

Elevated troponin levels are frequently observed in patients with HCM. Some studies have reported that high-sensitivity cardiac troponin is associated with LV remodelling and clinical outcomes [59, 60]. Elevated post-exercise troponin is frequently observed in patients with HCM (approximately 20%), and they also have high T2 values on CMR [61]. In another study, resting troponin was elevated in 50% of patients with HCM, with significantly higher levels in patients with angina than patients without angina symptom [62]. In patients with unclear symptoms but elevated troponin, a provocation test may be considered before starting exercise. However, as exercise-induced cardiac troponin elevation is also observed in the healthy population and the clinical implication of post-exercise troponin elevation has not been established, more research is still needed in this area [63].

## 6. Conclusions

Patients with HCM have highly heterogeneous morphology, functional status, pathophysiology, and prognosis. Therefore, either restricting their exercise or allowing them to exercise at will seems an inappropriate guide. Careful assessment of intrinsic substrates and estimation of the expected risk of adverse events are required. In addition, physicians should be aware of the characteristics associated with increased risk and the tests used to identify them. Finally, adjustments for exercise-related extrinsic factors and shared decision-making with patients are warranted to make final decisions.
